# A novel LC-MS/MS method combined with derivatization for simultaneous quantification of vitamin D metabolites in human serum with diabetes as well as hyperlipidemia

**DOI:** 10.1039/d3ra05700c

**Published:** 2023-11-22

**Authors:** Xiaodi Wang, Qian Qin, Fasheng Li, Ying Fu, Na Liu

**Affiliations:** a College of Medical Laboratory Science, Dalian Medical University Dalian Liaoning 116044 China liuna@dmu.edu.cn; b Department of General Surgery, Linyi People's Hospital Linyi Shandong 276600 China

## Abstract

Vitamin D plays an important role in calcium homeostasis. Recent studies indicate that vitamin D deficiency has become a major public health problem. In order to define vitamin D status, many analytical methods were used to quantify 25-hydroxyvitamin D (25OHD), as circulating 25OHD is regarded as the best indicator to evaluate vitamin D status. The current LC-MS/MS technology is internationally recognized as the “gold standard” for the detection of vitamin D and its metabolites. The impediment to the analysis of vitamin D metabolites is the low level of 25OHD and 1,25(OH)_2_D. Therefore, it is challenging to achieve the desired sensitivity and accuracy in the determination of trace vitamin D compounds in biological liquids. Here, a method based on liquid–liquid extraction in combination with derivatization, followed by liquid chromatography-electrospray/tandem mass spectrometry was developed for determination of the vitamin D metabolites, including 25-hydroxyvitamin D_2_, 25-hydroxyvitamin D_3_, 1α,25-dihydroxyvitamin D_2_ and 1α,25-dihydroxyvitamin D_3_. The method was simple and rapid, and it was validated with good linearity (*R*^2^ > 0.998), excellent recovery (average value with 81.66–110.31%) and high precision of intra-day and inter-day (0.06–6.38% and 0.20–6.82%). The values of limit of detection (LOD) and limit of quantitation (LOQ) were as low as 0.3 ng mL^−1^ and 1.0 ng mL^−1^, respectively. Finally, the developed method was successfully applied to determination of the vitamin D metabolites from the human serum samples of healthy subjects and patients with diabetes as well as hyperlipidemia.

## Abstract 2

A novel LC-MS/MS method combined with liquid–liquid extraction and derivatization was developed to analyze vitamin D metabolites and applied to the determination of the vitamin D metabolites in human serum with diabetes as well as hyperlipidemia.

## Graphical abstract

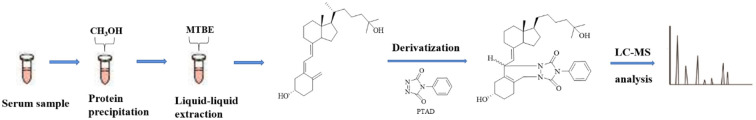

## 1. Introduction

Vitamin D plays a key role in bone health and calcium and phosphorus metabolism.^1–3^ In addition, it has a variety of biological functions, including inducing cell differentiation, inhibiting cell growth, immune regulation, *etc.*^4,5^ There are two main forms of vitamin D, including ergocalciferol (vitamin D_2_) and cholecalciferol (vitamin D_3_). Vitamin D_2_ and vitamin D_3_ are obtained from different sources. Vitamin D_3_ is mainly formed by ultraviolet B irradiation of 7-dehydrocholesterol under the skin but can also be obtained from dietary supplements, whereas vitamin D_2_ can be obtained from dietary supplements.^6,7^

**Fig. 1:**
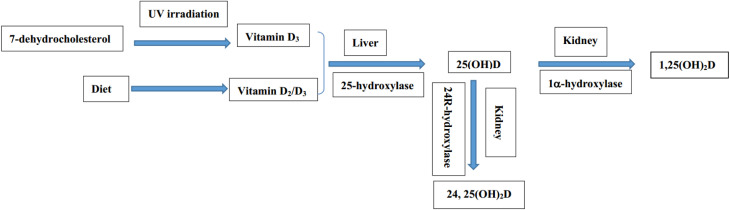
Schematic diagram of the biotransformation pathway of vitamin D and the related metabolites.

Due to the essential role of vitamin D in human health, several studies have reported that vitamin D deficiency is related to many diseases, including cancer, cardiovascular disease, autoimmune disease, schizophrenia, depression, *etc.*^8,9^ Especially, a growing number of studies have also found a strong link between vitamin D deficiency and diabetes and hyperlipidemia.^10–14^ Vitamin D has no biological activity and needs to be converted into bioactive metabolites through two steps of hydroxylation.^15,16^ The biotransformation pathway of vitamin D and the related metabolites is demonstrated in Fig. 1.

Quantification of 25OHD in human serum is the best index to evaluate vitamin D status.^17^ Various methods are used for the quantification of circulating 25OHD, including radioimmunoassay (RIA), enzyme-linked immunosorbent assay (ELISA) and high-performance liquid chromatography. Radioimmunoassay (RIA) and enzyme-linked immunosorbent assay (ELISA) are unable to separate 25OHD_2_ and 25OHD_3_.^17^ And it's reported that an optical biosensor is used to determinate vitamin D_3_, which is unable to analyze multiple analytes simultaneously.^18^ This indicates the need for selective analytical methods for vitamin D_2_ and vitamin D_3_ forms.^19^ The emergence of liquid chromatography-tandem mass spectrometry (LC-MS/MS) is based on the latest development of HPLC technology. Watson *et al.*^20^ first described LC-MS/MS method to measure vitamin D_2_, vitamin D_3_ and their respective monohydroxy and dihydroxy metabolites. This method has been greatly developed in clinical laboratories in the past 10–15 years, especially for quantitative analysis of low molecular weight analytes, such as vitamins, hormones and steroids. In recent years, owing to inherent specificity and sensitivity, LC-MS/MS has become a popular method and become the gold standard for the detection of vitamin D and its metabolites.^19^ This technique also has ability to simultaneously measure 25OHD_2_ and 25OHD_3_ and can separate them.^15^

Vitamin D metabolites have the low ionization efficiency due to the lack of easily charged groups in electrospray ionization (ESI) or atmospheric pressure chemical ionization (APCI) sources. Derivatization techniques have been developed to improve the detection response of low ionized compounds.^7,21^ However, the conjugated diene groups of vitamin D metabolites make it a specific target for Diels–Alder derivatization. 4-Phenyl-1,2,4-triazolin-3,5-dione (PTAD) reagent has been widely used in the synthesis of vitamin D compounds with s-*cis*-diene group. Derivatization reagents introduce polar groups, so the sensitivity of derivatization reagents is usually 10 times higher than that of non-derivatized compounds.^19,22^

In addition, the components of biological samples are too complex and contain more endogenous interference. When using this technology to analyze samples, the samples will pollute the instrument and the chromatographic column. If the sample extraction is not pure, it will produce a relatively high matrix effect, so the sample pretreatment technology process has attracted more and more attention of researchers.^23^ Generally, the pretreatment methods of samples include protein precipitation,^24^ liquid phase extraction,^25^ solid phase extraction^7^ and supported liquid–liquid extraction.^26^ Protein precipitation can release vitamin D metabolites which are closely bound with protein. Solid phase extraction (SPE) used to extract vitamin D metabolites from complex samples is labor-intensive and time-consuming, and resulting in low recovery with 59.1 and 60.2% for vitamin D_2_ and vitamin D_3_.^27^ While liquid–liquid extraction (LLE) is a classical and widely used technique in the analysis of vitamin D and its metabolites. The LLE method is simple and time-saving. The commonly used liquid–liquid extraction solvents are methyl *tert*-butyl ether, hexane, ether, ethyl acetate, chloroform or the mixture of non-polar solvents. In vitamin D analysis, LLE includes the addition and mixing of solvents with relatively small polarity and incompatible with water to extract hydrophobic vitamin D compounds. According to the subsequent analytical techniques, the organic layer is separated, the solvent is removed, and the sample is recombined in a suitable solvent.^25^

In this study, a highly sensitive method based on LLE and derivatization combined with LC-MS/MS was developed to achieve simultaneous analysis of the vitamin D metabolites. Initially, the vitamin D metabolites in biological sample were captured using LLE method. Then, a derivatization with high efficiency was carried out by introducing the derivative reagent of PTAD *via* Diels–Alder reaction. Next, a LC-MS/MS approach was used to analyze the derivatives of the vitamin D metabolites. The linearity, limit of detection (LOD), limit of quantitation (LOQ), matrix effects, recovery and precision were further investigated. Finally, the established method was used to the analysis of human serum vitamin D metabolites from healthy persons, diabetes and hyperlipidemia patients to prove its practicability and applications.

## 2. Materials and methods

### 2.1 Chemicals and reagents

25-Hydroxyvitamin D_2_ (25OHD_2_), 25-hydroxyvitamin D_3_ (25OHD_3_), 1,25-dihydroxyvitamin D_2_ [1,25(OH)_2_D_2_], 1,25-dihydroxyvitamin D_3_ [1,25(OH)_2_D_3_] and internal standard [26,26,26,27,27,27-2H6]-25-hydroxy vitamin D_3_ (25OHD_3_-d_6_) were purchased from Zhen Zhun company (Shanghai, China). The purity of the above standards is more than 98%. 4-Phenyl-1,2,4-triazole-3,5-dione (PTAD) was obtained from French Latin company (Shanghai, China). Acetonitrile (ACN, LC grade), methanol (MeOH, LC grade), methyl *tert*-butyl ether (MTBE) and ethyl acetate were purchased from J&K Scientific Ltd (Beijing, China). Formic acid (FA, analytic grade, 88%) was acquired form Kernel (Tianjin, China). Nitrogen (N_2_) was purchased from Da Te Gas Co, Ltd (Dalian, China). Ammonium hydroxide solution was purchased from Sigma-Aldrich (St. Louis, MO, USA).

### 2.2 Stock solutions preparation

Vitamin D metabolites were obtained as standard solutions in ethanol and they were diluted with methanol. 1 mg internal standard (25OHD_3_-d_6_) was dissolved in 1 mL methanol in the original vial and then transferred into an amber glass HPLC vial. The internal standard work solution containing 100 ng mL^−1^ 25OHD_3_-d_6_ in methanol was used for the calibration study. The vitamin D metabolites work solutions with concentrations of 1–100 ng mL^−1^ were prepared for standard curves. 10 mg PTAD was dissolved in 1 mL ethyl acetate with a concentration of 10 mg mL^−1^. All the solutions were stored in amber vials at −20 °C in the darkness until use.

### 2.3 Sample collection

In this study, the human serum samples from healthy people and patients with diabetes and hyperlipidemia were collected from Dalian Friendship People's Hospital. Before laboratory study, written informed consent was signed by all volunteers, and the research protocol (no. 2021(002)) was approved by the ethics committee of Dalian Medical University. The human serum samples without hemolysis, jaundice and cyclosis were frozen at −80 °C before use.

### 2.4 Sample pretreatment

**Fig. 2:**
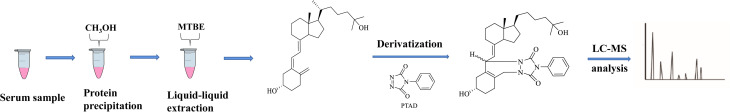
Schematic diagram of the vitamin D analysis process of human serum samples.

The human serum was thawed, and 100 μL aliquots of human serum in 1.5 mL EP tubes were spiked with 50 μL internal standard (25OHD_3_-d_6_) solution with a concentration of 200 ng mL^−1^ in methanol and allowed to equilibrate for 3 min at room temperature. The sample was added with 400 μL methanol to precipitate proteins, and then vortexed on a vortex mixer for 1 min, followed by centrifugation at 3500×*g* for 10 min. The supernatant was transferred into 1.5 mL EP tube and spiked with 400 μL MTBE and vortexed using a vortex mixer for 1 min, then centrifuged at 3500×*g* for 10 min, and subsequently the upper organic layer was transferred into 1.5 mL EP tube. The residue solution was added 200 μL MTBE and mixed using a vortex mixer for 1 min, and then centrifuged at 3500×*g* for 10 min. Finally, two extraction solutions were merged, and were dried by N_2_ gas stream. A 50 μL aliquot of 0.4 mg mL^−1^ PTAD in ethyl acetate was added to above dried sample with vortexing for 1 min, and then allowed to react at 60 °C for 10 min. In order to terminate the reaction, 50 μL ethanol was added to the mixture. The above mixed solution was evaporated to complete dryness under N_2_ gas stream. The dry residue was reconstituted with methanol–water (9 : 1, v/v) containing 0.1% FA, and centrifuged for 10 min at 10 000×*g* before transferring the supernatant into UHPLC vials. An aliquot (10 μL) was injected into the LC-MS/MS system for analysis. AA schematic diagram of the vitamin D metabolites analysis process of human serum samples was displayed in Fig. 2.

### 2.5 Optimization of solvents to precipitate protein

To investigate protein precipitation efficiency, ACN and CH_3_OH were commonly used as protein precipitation solvents. For each protein precipitation, 400 μL of individual solvent was added to 100 μL of human serum spiked with mixed standard solution at the concentration of 500 ng mL^−1^. And the above mixture was vortexed for 1 min. The remaining steps were the same as that described in Section 2.4. According to the signal response intensity of the vitamin D metabolites, the best protein precipitation solvent was selected.

### 2.6 Optimization of extraction conditions

In order to extract as much vitamin D metabolites as possible, common extraction solvents, including methyl *tert*-butyl ether (MTBE) and chloroform-methanol (CHCl_3_–CH_3_OH, v/v = 2 : 1) were examined. For each extraction, 400 μL of individual solvent was added to the remaining solution after protein precipitation, and the mixed solution was vortexed for 1 min, followed by centrifugation at 3500×*g* for 10 min. The remaining steps were the same as Section 2.4. In addition, the extraction times was also investigated. Six replicated samples were prepared, each three was in a group. Experimental steps of the first group were carried out as the same as Section 2.4, while the experimental steps of the second group were different from those of the first group only in the extraction times. The second group was extracted with MTBE after protein precipitation and then dried with nitrogen streams. The other steps were consistent with those described in Section 2.4.

### 2.7 Optimization of derivatization conditions

The molar weight of the derivatization reagent has a key effect on the derivative reaction. In order to make the derivatization reagent to react with vitamin D metabolites completely in human serum samples, the ratio of the derivative substance to the standard substance with a certain molar amount was investigated to select the optimal molar amount of the derivatization reagent. 18 EP tubes divided into six groups were prepared, 100 μL 100 ng mL^−1^ vitamin D metabolites mixed standard solution was added to each EP tube, then the PTAD at six different concentrations were added. The molar ratios of the derivatization reagent to the standard were 2, 20, 200, 2000, 9000, and 12 000. The mixtures were vortexed for 1 min, and then incubated in a water bath at 60 °C for 10 min. The excess PTAD was reacted with 50 μL ethanol. Then the mixtures were vortexed for 1 min and centrifuged at 10 000×*g* for 10 min. Subsequently, 200 μL of supernatant was transferred to an HPLC vial and analyzed using LC-MS/MS. Finally, the corresponding signal intensities were compared. Moreover, for the sake of improving derivatization efficiency, the derivatization reaction time was optimized. 1.5 mL 100 ng mL^−1^ vitamin D metabolites mixed standard solutions were prepared, 100 μL 0.4 mg mL^−1^ PTAD was added into each tube. The above mixture was vortexed for 1 min, and then incubated in a water bath at 60 °C for 5 min, 10 min, 20 min, 40 min, and 60 min, respectively. 50 μL ethanol was added to terminate the reaction. After vortexed for 1 min and centrifuged at 10 000×*g* for 10 min, 200 μL of the supernatant was transferred into an HPLC vial and analyzed by LC-MS/MS. The corresponding signal intensities were compared.

### 2.8 Optimization of redissolved solvents

Prior to LC-MS/MS analysis, it is essential to redissolve the dried samples. According to the polarity of vitamin D, methanol, ethanol and acetonitrile were selected as the redissolving solvent. The dried sample was redissolved with methanol, ethanol and acetonitrile, respectively. A certain proportion of water was added to improve the ionization strength. After the sample was dried in nitrogen streams, the sample was dissolved with the above-mentioned proportion of solvent, the mixed solution was vortexed for 1 min, followed by centrifugation at 3500×*g* for 10 min. Then the samples were analyzed by LC-MS/MS. The corresponding signal intensities were compared, and the group of redissolved solvents with better effect was selected.

### 2.9 LC-MS/MS analysis

The LC-MS analysis was performed with an Agilent 1290 UHPLC system, coupled with an API 3200 MS/MS system (Applied Biosystems SCIEX, USA). An ACQUITY UPLC separation module was used for separation. The UPLC was carried out on a CSH C18 column (2.1 mm × 100 mm, 1.7 μm, Waters Corporation, USA). The column temperature was kept at 50 °C. Samples were eluted at a flow rate of 0.3 mL min^−1^ using binary phases. Phase A was water (0.1% formic acid and 10 mM ammonium acetate) and phase B was methanol (0.1% formic acid and 10 mM ammonium acetate). The linear gradient conditions were as follows: 0 min, 98% B; 1 min, 98% B; 2 min, 65% B; 4–7 min, 100% B; 10 min, 2% B; 12 min, 2% B. The total chromatographic run time was 12 min. The injection volume was 10 μL.

Mass spectrometer conditions were set as follows: CUR at 25 psi, GS1 at 45 psi, GS2 at 45 psi, IS at 5500 V, and TEM at 500 °C. The mass spectrometer was auto-equilibrated for 3 min. According to the structural characteristics of vitamin D metabolites, positive ion mode was used. The mass spectrometer was performed in the multiple reaction monitoring (MRM) mode. Diluted standards were used to optimize delustering potential (DP), entrance potential (EP), collision potential (CEP), Collision chamber outlet voltage (cell exit potential, CXP) and collision energies (CE). The acquisition parameters for vitamin D analytes were listed in Table 1.

**Table 1:** MRM parameters of the derivatives of the four vitamin D metabolites and internal standard^a^

Analytes	Precursor/product (Da)	DP (V)	EP (V)	CEP (V)	CE (V)	CXP (V)	Ion mode	Retention time (RT)
25(OH)D_2_	570.1/298.0	60	6	10	30	6	+	6.44
25(OH)D_3_	558.5/298.1	60	5	8	25	12	+	6.40
1,25(OH)_2_D_2_	586.3/314.2	60	5	7	28	6	+	6.23
1,25(OH)_2_D_3_	574.2/314.2	60	6	12	22	13	+	6.18
25(OH)D_3_-d_6_	564.2/298.2	60	5	8	23	13	+	6.38

a DP: delustering potential; EP: entrance potential, the inlet voltage that can direct and focus ions through the high-voltage Q0 region; CEP: collision entrance potential; CE: collision energy; CXP: cell exit potential.

### 2.10 Method validation

In order to check the reliability of the established method, the linearity, limit of detection (LOD), limit of quantitation (LOQ), matrix effect, recovery and intra-day/inter-day precision were investigated. For the purpose of analysis of extraction recovery and matrix effect, three groups of samples were prepared. The vitamin D metabolites were added to human serum samples to form three spiked samples at low concentrations of 1, 10, 5, 5 ng mL^−1^, medium concentrations of 5, 50, 10, 10 ng mL^−1^, and high concentration of 20, 100, 20, 20 ng mL^−1^ for 25OHD_2_, 25OHD_3_, 1,25(OH)_2_D_2_ and 1,25(OH)_2_D_3_, respectively. Similarly, the standard samples were prepared at the same concentrations as above and blank samples were individually prepared. A certain concentration of deuterium isotope internal standard was added to each group of samples to minimize the matrix effect.

The matrix effect was calculated by the following formula:

1 *R* (%) = 100 × (*S*_3_ − *S*_2_)/*S*_1_

The formula for calculating the recovery is as follows:

2 *R* (%) = 100 × (*S*_3_ − *S*_2_)/*S*_0_

where *S*_0_ referred to the relative peak area of vitamin D metabolites under the corresponding concentration standard curve, *S*_1_, *S*_2_ and *S*_3_ represented the peak area of vitamin D metabolites in standard solutions, blank human serum samples and spiked human serum samples *via* the same pretreatment conditions, respectively.

Linear regression of the standard concentrations on the theoretical peak area ratios of the target vitamin D metabolites and its corresponding internal standard was used to evaluate linearity. The intra-day precision was estimated by measuring five times in a single day, while the inter-day precision was estimated by measuring five times for 3 consecutive days.

## 3. Results and discussion

### 3.1 Optimization of deproteinized solvent

**Fig. 3:**
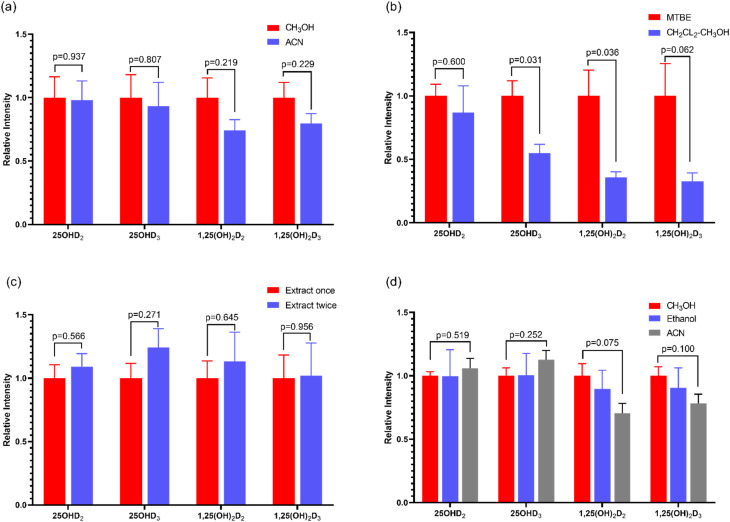
MS intensity of vitamin D metabolites with protein precipitation by CH_3_OH and ACN (a), extracted by MTBE and CH_2_Cl_2_–CH_3_OH (b), extracted by MTBE for once and twice (c), and redissolved in CH_3_OH, ethanol and ACN (d).

Protein precipitation is a common method used to release vitamin D metabolites from human serum biding proteins. In this study, methanol and acetonitrile were selected as two commonly used protein precipitation reagents for protein precipitation^28^ and the protein precipitation effects of the two groups were compared. The results were shown in Fig. 3a. The corresponding intensity of each vitamin D metabolite was 100% when deproteinized with methanol, while the corresponding intensity of each vitamin D metabolite when deproteinized with acetonitrile was a percentage of the corresponding strength of methanol. The results showed that the intensity of vitamin D metabolites was higher than that of acetonitrile when using methanol for protein precipitation detection, showing a higher extraction efficiency with methanol. Because of the hydroxyl group, 25OHD and 1,25(OH)_2_D have good solubility in methanol. Finally, methanol was selected to precipitate protein for detection of vitamin D metabolites in human serum.

### 3.2 Evaluation of the extraction approach

Through the investigation of the signal intensity of the compounds tested, the extraction effects of two vitamin D metabolites with different extraction solvents were compared. The results were displayed in Fig. 3b. The signal intensity of extraction with MTBE method was significantly higher than that of CH_2_Cl_2_–CH_3_OH (2 : 1, v/v). In addition, the extraction times were also optimized. Only one extraction may not be complete, so MTBE was used for the secondary extraction for vitamin D metabolites. Results in Fig. 3c, 25OHD_2_ and 25OHD_3_ showed obvious difference, that was, the efficiency of the twice extraction was higher than that of one extraction. However, there was no difference between the effect of 1,25 (OH)_2_D_2_ and 1,25 (OH)_2_D_3_ with extraction once or twice, which may be due to the very low content of 1,25 (OH)_2_D in human body, only in the range of pg mL^−1^. As a result, MTBE was selected as the extraction solvent of vitamin D metabolites in human serum. And it was used to extract twice.

### 3.3 Evaluation of derivatization approach

**Fig. 4:**
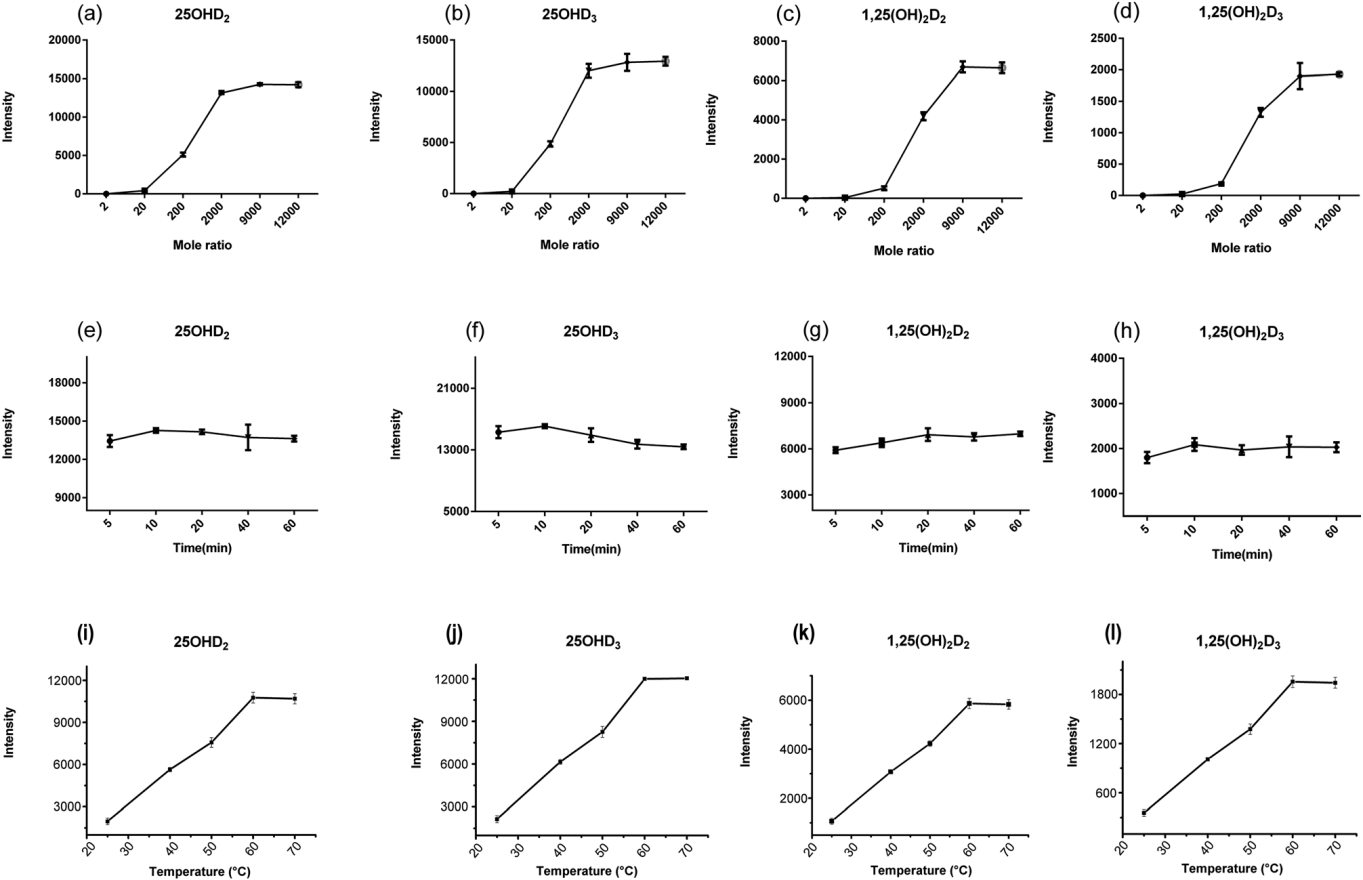
The optimization of the amount of derivative reagent (a–d), derivatization time (e–h) and derivatization temperature (i–l) for the vitamin D metabolites.

Some studies have shown that PTAD-derivatization might improve the ESI-MS/MS response of vitamin D metabolites, thus the quite low abundant analytes in biological fluids was allowed to be detected.^29^ In our study, the influence of the molar amount of PTAD on the signal intensity of vitamin D metabolites was investigated, and the optimal molar amount of PTAD was selected. The results were shown in Fig. 4a–d, the ratio of the molar amount of PTAD to that of the standard is 9000 As the molar amount of PTAD increases, the signal response intensity does not change much, which was almost the same as reported in many studies. Moreover, some studies have clarified that if the molar amount of PTAD is too high, it will inhibit the rate of derivatization reaction. The derivatization time from 5 to 60 min was also examined, as is shown in Fig. 4e–h. It is indicated in previous studies that derivatization time was 30–60 min.^7^ There are even 24 hours long for the derivatization reaction in some studies. Besides, the derivatization temperature has a key effect on the derivatization between vitamin D metabolites and PTAD. It can be seen from Fig. 4i–l, the derivatization reaction was more completed with the increase of temperature from 25 °C to 70 °C. When the derivatization temperature was at 60 °C, almost all vitamin D metabolites were derivatized. Further increase of temperature would be unfavorable to the stability of vitamin D metabolites. Thus, the derivatization for the vitamin D metabolites was carried out at 60 °C. It is indicated in this study that the optimum conditions for derivatization were PTAD at 0.4 mg mL^−1^ in ethyl acetate and an incubation time of 10 min at 60 °C. In our study, the completion time of the derivatization reaction is shorter, saving a lot of pretreatment time. This also shows that the rate of the derivatization reaction is fast and the conditions of the derivatization reaction are mild.

**Fig. 5:**
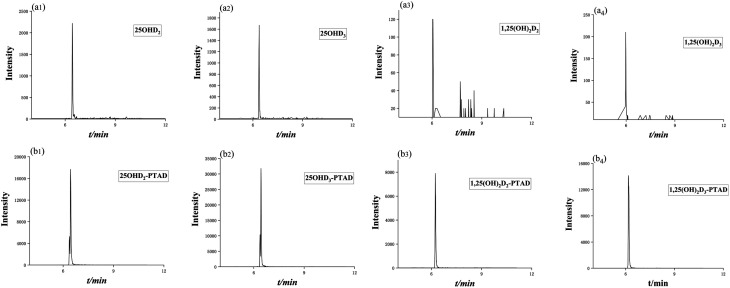
Mass chromatogram response of vitamin D metabolites before (a) and after derivatization (b).

After derivatization, the sensitivity of the vitamin D metabolites was increased by 10 to 66 times. The mass chromatogram response of the metabolites before and after derivatization with the derivatization reagent PTAD was shown in Fig. 5a and b, respectively.

### 3.4 Optimization of redissolving solvents

In order to dissolve the detected vitamin D as much as possible, the solvent with good solubility was selected for redissolution. In this study, methanol, ethanol and acetonitrile were selected as the solvents. According to the signal response intensity of the sample after redissolution, the optimal solvent was selected. The results were shown in Fig. 3d. For 25OHD_2_ and 25OHD_3_, the solubilization effect of acetonitrile was better than that of methanol and ethanol. The properties of methanol and ethanol were similar, and the solubility of vitamin D metabolites in these two solvents was similar. However, for 1,25(OH)_2_D_2_ and 1,25(OH)_2_D_3_, methanol is more soluble than 1,25(OH)_2_D_3_. Due to the low content of 1,25(OH)_2_D_2_ and 1,25(OH)_2_D_3_*in vivo*, it is difficult to be detected. Therefore, methanol was selected as the redissolving solvent. When the ratio of methanol to water was 9 : 1, the response intensity of vitamin D was higher.

### 3.5 Validation of the method

#### 3.5.1 Linear range, LOD and LOQ

The linearity of vitamin D metabolites was generated by plotting the peak area ratio (analyte/internal standard) *versus* six levels of concentrations. Co-mingled calibration standards were prepared at different levels in methanol. Internal standard solution was prepared in methanol at a concentration of 100 ng mL^−1^. The calibration curve was composed of the ratio of the chromatographic peak area of the substance measured and the chromatographic peak area of the internal standard substance. As summarized in Table 2, good linearities were acquired over wide concentration ranges with 1–100 ng mL^−1^ for 25OHD_2_ and 25OHD_3_, 5–100 ng mL^−1^ for 1,25(OH)_2_D_2_ and 1,25(OH)_2_D_3_, and the correlation coefficients (*R*^2^) were more than 0.998. The limits of detection (LOD) were 0.3, 0.3, 1.5 and 1.5 ng mL^−1^ for 25OHD_2_, 25OHD_3_, 1,25(OH)_2_D_2_ and 1,25(OH)_2_D_3_, respectively. The limits of quantification (LOQ) were 1.0, 1.0, 5.0 and 5.0 ng mL^−1^ for 25OHD_2_, 25OHD_3,_ 1,25(OH)_2_D_2_ and 1,25(OH)_2_D_3_, respectively.

**Table 2:** The linear range, *R*^2^, LOD and LOQ for the derivatives of the four vitamin D metabolites

Compounds	Linear equation	Linear range (ng mL^−1^)	*R* ^2^	LOD (ng mL^−1^)	LOQ (ng mL^−1^)
25(OH)D_2_	*Y* = 0.0076*x* + 0.0022	1–100	0.9999	0.3	1.0
25(OH)D_3_	*Y* = 0.0073*x* + 0.0181	1–100	0.9996	0.3	1.0
1,25(OH)_2_D_2_	*Y* = 0.0039*x* − 0.0008	5–100	0.9984	1.5	5.0
1,25(OH)_2_D_3_	*Y* = 0.0014*x* − 0.0013	5–100	0.9995	1.5	5.0

#### 3.5.2 The matrix effects, recovery and precision

To evaluate the reliability of the developed method, the matrix effects, recovery and intra-day/inter-day precision were further investigated. As listed in Table 3, the matrix effects of the derivatives of vitamin D metabolites at low, medium, high levels were 67.55–91.41%, 73.25–111.13% and 64.68–95.88%, respectively. And the coefficient of variations (CVs) of all the analytes at three concentration levels were in the range of 1.52–11.01%. It indicated that the matrix effect produced by endogenous matrix was acceptable. The recovery is mainly to determine whether the established sample treatment method could completely extract the components to be analyzed in the sample. The average recoveries of the derivatives of vitamin D metabolites were 81.66–110.31%. And the CVs of all the analytes at three levels were in the range of 0.91–13.95%. The relative standard deviations (RSDs) of intra-day precision were 2.92–11% and the RSDs of inter-day precision were 0.20–6.82%. These results are satisfying and acceptable, suggesting the method has a good accuracy.

**Table 3:** Matrix effect, recovery, intra- and inter-day precision of analytes in human serum

Spiking analytes	Concentration (ng mL^−1^)	Matrix effect (*n* = 3)		Recovery (*n* = 3)		Intra-day (*n* = 5)	Inter-day (*n* = 15)
		Mean ± SD	CV (%)	Mean ± SD	CV (%)	RSD (%)	RSD (%)
25OHD_2_	1	67.55 ± 3.94	3.82	124.11 ± 4.31	3.65	10.74	0.20
	5	111.13 ± 9.73	8.53	105.63 ± 13.48	12.10	6.15	0.20
	20	91.16 ± 1.25	1.52	101.19 ± 4.56	5.63	2.92	3.39
25OHD_3_	10	75.61 ± 8.52	10.22	96.78 ± 4.44	4.10	6.10	0.40
	50	101.95 ± 3.53	3.30	100.43 ± 2.18	1.96	4.51	6.82
	100	95.88 ± 6.91	5.48	103.65 ± 4.07	5.49	3.78	2.80
1,25(OH)_2_D_2_	5	91.41 ± 2.40	2.16	106.13 ± 0.97	0.91	11.01	0.96
	10	73.25 ± 6.12	7.18	125.24 ± 1.90	1.49	11.22	2.61
	20	72.57 ± 4.75	5.99	85.69 ± 11.36	11.34	5.26	5.26
1,25(OH)_2_D_3_	5	89.19 ± 6.40	6.43	76.12 ± 3.84	6.24	11.46	2.18
	10	76.02 ± 3.30	3.02	96.84 ± 9.74	13.95	10.37	2.58
	20	64.68 ± 7.44	11.01	72.03 ± 5.35	8.32	7.00	2.61

#### 3.5.3 Comparison with previously reported methods

The results obtained from the developed method using LC-MS/MS combined with derivatization was compared with other previously reported methods^18,30–32^ as presented in Table 4. The developed method has high sensitivity. And compared with other reported method with SPE or LLE combined with SPE,^32,33^ only LLE pretreatment method used in this study is generally relatively simple to operate and requires more common equipment and reagents. Besides, compared with the reported method with 0.5–1 h for derivatization time,^7^ only 10 min for derivatization time was needed in this work, saving pretreatment time, which is very conducive to high throughput analysis.

**Table 4:** Comparison of the developed method *versus* some previously reported methods for determination of vitamin D_2_ and vitamin D_3_

Analyte	Methods	Linearity	Limit of detection	References
Vitamin D_2_ [25(OH)D_2_]	LLE + derivatization + LC-MS/MS	1.5–48 nmol L^−1^	0.3 nmol L^−1^	Ref. 30
	HPLC-MS/MS	3.9–183.6 nmol L^−1^	3.9 nmol L^−1^	Ref. 31
	LLE + derivatization + LC-MS/MS	1–100 ng mL^−1^	0.3 ng mL^−1^	This work
Vitamin D_3_ [25(OH)D_3_]	Spectrofluorometric using Tb^3+^-ACV	1.0–320 nmol L^−1^	1.19 nmol L^−1^	Ref. 18
	LLE + derivatization + LC-MS/MS	7.8–250 nmol L^−1^	1.5 nmol L^−1^	Ref. 30
	HPLC-MS/MS	4.0–265.3 nmol L^−1^	4.0 nmol L^−1^	Ref. 31
	SPE + LC-MS/MS	1–60 ng mL^−1^	1 ng mL^−1^	Ref. 32
	LLE + derivatization + LC-MS/MS	1–100 ng mL^−1^	0.3 ng mL^−1^	This work

### 3.6 Determination of vitamin D metabolites in human serum samples from healthy subjects and patients with diabetes and hyperlipidemia

**Fig. 6:**
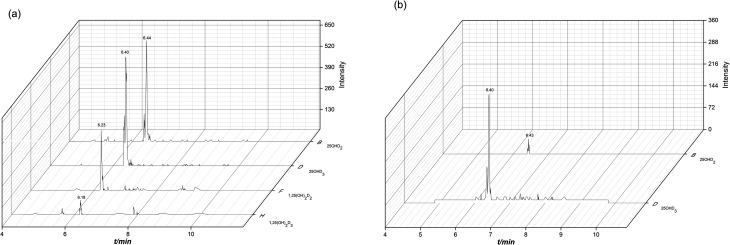
MRM chromatograms of the derivatives of the vitamin D metabolites obtained from standard mixture (a) and the human serum sample (b).

**Fig. 7:**
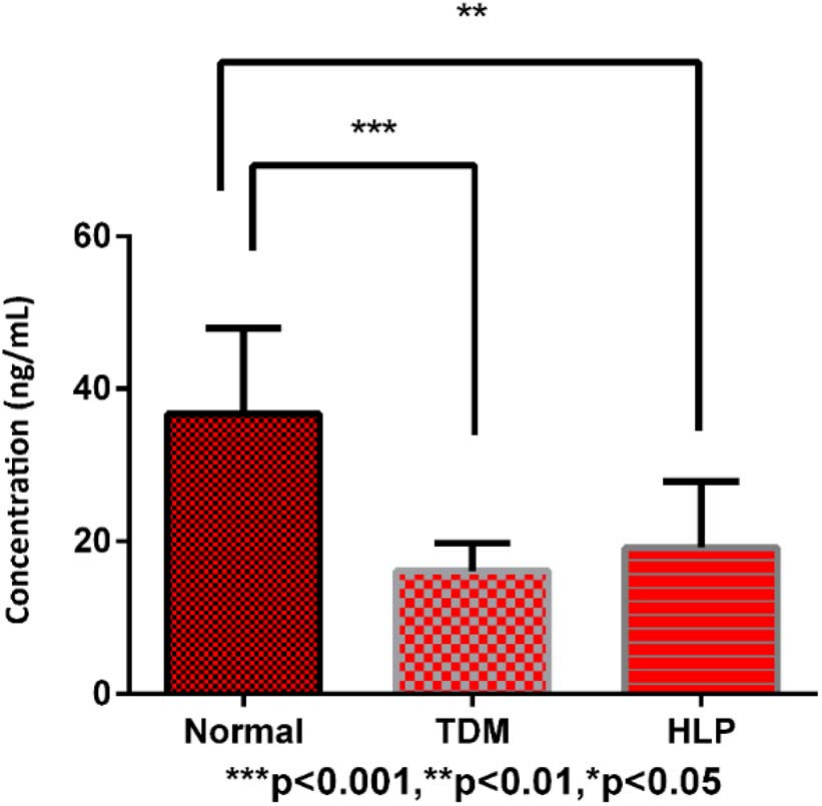
Univariate analysis of the vitamin D metabolites from normal subjects, diabetes (TDM) and hyperlipidemia (HLP) patients.

As an endogenous substance, vitamin D metabolites may have significant variation and disorder in healthy people and some metabolic diseases. The developed derivatization and LC-MS/MS method was applied to analyze the vitamin D metabolites in human serum samples of healthy people (*n* = 10), diabetes (*n* = 10) and hyperlipidemia (*n* = 10) patients. Among all the vitamin D metabolites, 25OHD_2_ and 25OHD_3_ could be detected by using the established method. The MRM chromatograms of the vitamin D metabolites from the standard mixture and the human serum sample were shown in Fig. 6a and b. As a result, the concentrations of 25OHD_2_ in the human serum samples from 10 healthy people, 10 diabetes and 10 hyperlipidemia patients were 1.00–7.80 ng mL^−1^, 1.10–4.04 ng mL^−1^ and 1.00–2.32 ng mL^−1^, respectively. And the concentrations of 25OHD_3_ in the above three groups were 19.66–52.30 ng mL^−1^, 9.86–20.83 ng mL^−1^ and 10.00–41.00 ng mL^−1^, respectively. The contents of vitamin D metabolites in human serum determined in this study were in accordance with the order of magnitude of the contents in the literatures.^23,24^ Univariate analysis showed that total contents of 25OHD_2_ and 25OHD_3_ had significant difference (*p* < 0.01) between normal subjects and patients with diabetes as well as hyperlipidemia (Fig. 7). Most experts believed that the concentration of 25OHD below 20 ng mL^−1^ (50 nmol L^−1^) were signs of vitamin D deficiency, while the concentration with 21–29 ng mL^−1^ (51–74 nmol L^−1^) were considered to be signs of vitamin D insufficiency.^28^ Most studies in the literature showed that the content of 25OHD in patients with diabetes mellitus was deficient.^34^ Vitamin D insufficiency may contribute to the pathogenesis of diabetes, and epidemiological evidence suggests that it is associated with insulin resistance.^35^ The content of 25OHD measured in this study was in the range of deficiency in patients of diabetes, which is in line with expectations.^34^ The content of 25OHD measured in patients with hyperlipidemia in this study was also in the range of deficiency. It was reported that vitamin D deficiency is associated with hyperlipidemia, and elevated human serum 25OHD levels were associated with decreased human serum TC and TG levels, especially in patients with hyperlipidemia who were vitamin D deficiency.^14^ Therefore, increasing the concentration of circulating 25OHD may be used as an adjunct therapy for hyperlipidemia.^14^ It has a certain reference value for clinical diagnosis and treatment.

## 4. Conclusions

In this study, a novel method based on liquid–liquid extraction, derivatization and LC-MS/MS was established to simultaneously analyze the vitamin D metabolites. PTAD was used to derivatize the vitamin D metabolites. The sample pretreatment process of the method was simple and rapid, and the derivatization reaction conditions were mild. Moreover, the method with good linearity, precision, recovery and sensitivity was reliable to analyze the vitamin D metabolites in real samples. The method showed good practicability in studying the vitamin D metabolites in relation to metabolic diseases. The results indicated that there were significant differences in human serum 25OHD levels between healthy individuals, diabetes and hyperlipidemia patients. Consequently, the developed method was useful to determine the vitamin D metabolites, and it's promising to apply it to the research of other vitamin D metabolites-related diseases.

## Conflicts of interest

There are no conflicts to declare.

## Supplementary Material
